# An unusual presentation of purine nucleoside phosphorylase deficiency mimicking systemic juvenile idiopathic arthritis complicated by macrophage activation syndrome

**DOI:** 10.1186/s12969-019-0328-3

**Published:** 2019-05-22

**Authors:** Alessia Arduini, Emiliano Marasco, Giulia Marucci, Manuela Pardeo, Antonella Insalaco, Ivan Caiello, Gian Marco Moneta, Giusi Prencipe, Fabrizio De Benedetti, Claudia Bracaglia

**Affiliations:** 1 Pediatric Department, La Sapienza University of Rome, Rome, Italy; 2 Division of Rheumatology, IRCCS Ospedale Pediatrico Bambino Gesù, Piazza S. Onofrio 4, 00165 Rome, Italy

**Keywords:** Macrophage activation syndrome, Juvenile idiopathic arthritis, Interferons, Interleukins, Chemokines

## Abstract

**Background:**

Systemic juvenile idiopathic arthritis (sJIA) is an inflammatory condition that presents with fever, rash and arthritis. At onset systemic features are predominant and the diagnosis may be a challenge. Secondary hemophagocytic lymphohistiocytosis (sHLH) forms may be associated with different disorders, including rheumatic diseases, and this form is called macrophage activation syndrome (MAS). CXCL9 levels, a chemokine induced by IFNγ, are significantly elevated in patients with sHLH or MAS and are correlated with laboratory features of disease activity. High levels of IL-18 have been reported in patients with MAS during sJIA, as well as in some patients with sHLH and IL-18 is indeed known to induce IFNγ production.

**Findings:**

We report a patient with a clinical presentation highly suggestive for systemic juvenile idiopathic arthritis (sJIA) onset complicated by MAS, and was later diagnosed with purine nucleoside phosphorylase (PNP)-deficiency with HLH. Some unusual features appeared when HLH was controlled and further investigations provided the correct diagnosis. Serum CXCL9 and IL-18 levels were found markedly elevated at disease onset, during the active phase of MAS and decreased progressively during the course.

**Conclusion:**

The reported case underlines the potential difficulties in discriminating sJIA from other causes of systemic inflammation. Furthermore, this supports the notion that especially in young children with a sJIA-like disease other mimicking conditions should be actively sought for. CXCL9 and IL-18 levels suggested that patients with PNP-deficiency may have a subclinical activation of the IFNγ pathway and indeed they are predisposed to develop sHLH.

## Findings

The onset of systemic juvenile idiopathic arthritis (sJIA) is characterized by quotidian fever associated with rash and arthritis. Arthritis may become evident later. Laboratory features of sJIA are not specific and include marked increase in acute-phase reactants and in platelet and white blood cell counts. The onset of sJIA may also be complicated by occurrence of macrophage activation syndrome (MAS). MAS, a severe, potentially fatal, complication of sJIA, is classified among the secondary forms of haemophagocytic lymphohistiocytosis (HLH), as an HLH occurring in the context of a rheumatic disease [1, 2].

Other secondary HLH (sHLH) forms may be associated with infections, malignancy or metabolic disorders. Primary HLH are caused by mutations of genes coding for proteins involved in cytotoxic activity or occur in the context of immunodeficiency. In a significant number of cases an underlying trigger cannot be found [3].

## Methods

Serum chemokine (C-X-C motif) ligand 9 (CXCL9) levels and interleukin-18 (IL-18) levels were measured with ELISA (DuoSet ELISA KIT, R&D Systems, Minneapolis, Minnesota and Medical and Biologic Laboratories, Nagoya, Japan, respectively).

Peripheral blood mononuclear cells (PBMCs) were isolated with Ficoll density gradient centrifugation. Lymphocytes subsets analysis was performed by cytometry [4]. To assess T cell proliferation, 10^6^/mL PBMCs were cultured with 1.5 μg/mL of anti-CD3 (clone OKT3) or with 4 μg/mL of phytohemoagglutinin (PHA) for three days. Proliferation was assessed with a standard [^3^H]-thymidine incorporation assay. Degranulation of NK and T cells was assessed incubating PBMCs with K562 cells overnight followed by measurement of the translocation of CD107a to the cell surface by flow cytometry.

Parents provided an informed consent. The local Institutional Ethical Committee approved the study (number 1649/2018).

## Results

**Fig. 1:**
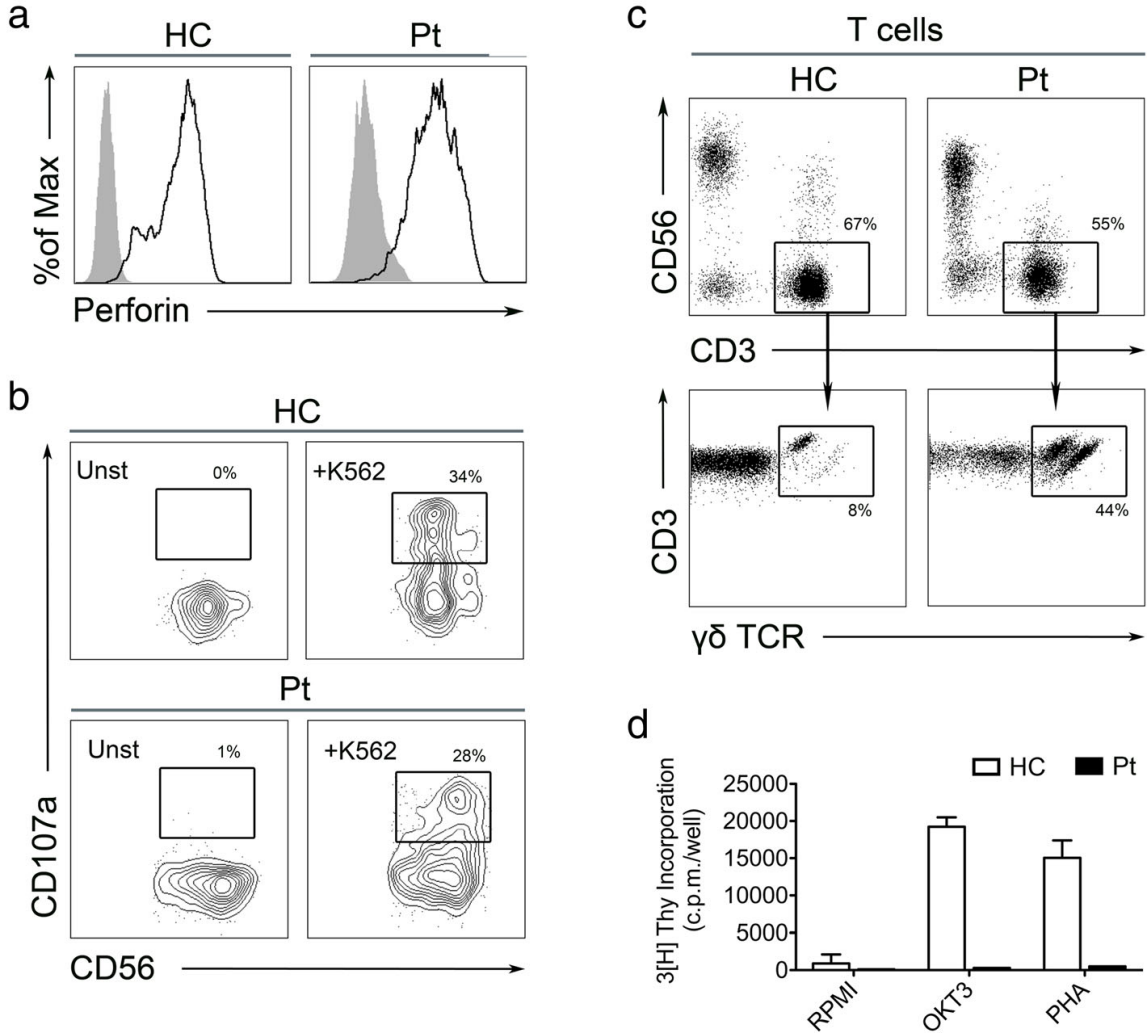
Immunological functional tests of the patient. Intracellular expression of perforin in CD56^+^ NK cells was assessed by flow cytometry (**a**). Degranulation potential of CD56^+^ NK cells was assessed by flow cytometry after overnight co-culture with target cells K562. Plots show the surface expression of CD107a on unstimulated and stimulated NK cells (**b**). Lymphocytes subsets were analysed by flow cytometry: T cells were identified as CD3^+^ CD56^−^ CD19^−^; within the CD3^+^ gate, γδ-T cells were gated as CD3^+^ γδ-TCR^+^ αβ-TCR^−^ (**c**). T cell proliferation was assessed by a standard [^3^H]-thymidine incorporation assay following stimulation with anti-CD3 (OKT3) or PHA (**d**). The results are shown for first determination while on 2 mg/Kg of methylprednisolone. They were repeated on a lower dose of oral prednisone (0.25 mg/Kg) with similar results

A 14 months-old Caucasian male, born by vaginal delivery, (normal birth weight and normal APGAR score) from non-consanguineous parents, was admitted with persistent fever of 2 weeks duration, palpable cervical lymph nodes, rash, pericardial effusion and arthralgia. On suspicion of Kawasaki disease he had been treated with two doses (2 g/kg) of intravenous immunoglobulins without response. The neonatal period was regular, he had not infection, growing normally. At age of 7 months, the paediatrician observed a neurological delay, no investigation was performed. At first examination, he appeared generally unwell with a maculopapular rash on face and legs. Cardiovascular, respiratory and abdominal were unremarkable. He developed overt swelling of wrists and ankles, with evidence of tenosynovitis on ultrasound. CRP and ESR were elevated. Leukopenia with lymphopenia and anaemia were present. Serum ferritin, lactate dehydrogenase, aspartate aminotransferase and triglyceride levels were markedly elevated (Table 1). Bone marrow biopsy showed hemophagocytosis. No infections were detected. Chest X-ray was negative. Echocardiography showed a pericardial effusion. Abdominal ultrasound did not reveal splenomegaly or hepatomegaly. Based on the presence of arthritis, with tenosynovitis, in a child with fever, rash and pericarditis, a diagnosis of sJIA was made, with onset complicated by MAS. Treatment with glucocorticoid (3 pulses of methylprednisolone 30 mg/kg/day), followed by oral prednisone (2 mg/kg/die), and anakinra (4 mg/Kg/die) was started with progressive improvement. Perforin expression and CD107 degranulation test were normal (Fig. 1a, b), sIL2 receptor was not measured; genetic analysis of the primary HLH related genes (*PRF1*, *RAB27a*, *SH2D1A*, *STX11*, *STXBP2*, *XIAP*, *UNC13D*, *LYST*) was negative.

**Table 1:** Laboratory parameters and cytokine levels during disease course

Laboratory parameters	Onset	2 weeks after HLH-treatment was started	6 weeks before HSCT	12 months after HSCT
White blood counts (6-17 × 10^3^/uL)	3.4	14.54	5.4	10.1
Neutrophils (1.68–8.5 × 10^3^/uL)	2.33	12.51	3.86	4.3
Lymphocytes (3–11.2 × 10^3^/uL)	0.5	0.34	0.62	8.51
Haemoglobin (10.5–15.5 g/dl)	7.1	8.0	8.0	10.9
Platelet (150-450 × 10^3^/uL)	360	377	406	393
Ferritin (17–390 ng/ml)	24,716	25	2051	34
Fibrinogen (200–500 mg/dl)	253	240	219	208
D-dimer (< 0.5 microg/ml)	2.81	0.35	0.5	0.25
PTT (25–34 s)	27.3	25.2	33	31.9
PT (12.1–14.5 s)	13	13.4	12	13.3
PT RATIO-INR (0.92–1.14 s)	1.07	1.13	0.92	1.07
Triglycerides (40–150 mg/dl)	247	111	316	110
AST (5–40 UI/L)	97	40	48	32
ALT (5–40 UI/L)	27	18	14	34
LDH (100–600 UI/L)	2217	525	869	385
CRP (0–0.5 mg/dl)	4.18	< 0.05	0.07	0.05
IL-18 (pg/ml)^a^	250,000	83,200	113,789	1024
CXCL9 (pg/ml)^a^	3556	4942	3327	< 300

*AST* aspartate aminotransferase, *ALT* alanine aminotransferase, *LDH* lactate dehydrogenase, *CRP* C-reactive protein, *HSCT* hematopoietic stem cell transplantation

^a^The values for IL-18 and CXCL9 established in healthy controls are: mean 265.0 pg/ml, IQR 221.3–305.1 pg/ml for IL-18 and 612.4 pg/ml, IQR 429.5–801.5 pg/ml for CXCL9

After two weeks, laboratory parameters normalized except for persistent anaemia (Hb 8.9 g/dl) and variable lymphopenia (500–900/μL). Moreover, a mild muscle hypotonicity became apparent confirmed by neurological evaluation. Additional laboratory investigations revealed reduced haptoglobin (< 1 mg/dl), high reticulocyte count (9.34%) and positive Coombs test, all suggestive of haemolytic anaemia. Immunological phenotyping revealed a normal frequency of NK cells (33%), B cells (5%) and T cells (53%), whereas absolute numbers of CD4^+^ (185 cells/μL) and CD8^+^ (211 cells/μL) T cells were reduced. The T cell compartment showed expansion of a subpopulation of CD3^+^CD4^−^CD8^−^ expressing the γδ-TCR (no expansion of CD3^+^CD4^−^CD8^−^ αβ-TCR was observed) and absence of proliferative response to PHA and OKT3 (Fig. 1c, d). A metabolic screening showed increase in urine orotic acid (50.2 M/mM), with normal citrulline serum level (25 micromol/l) and low serum level of uric acid (0.1 mg/dl). Based on the presence of haemolytic anaemia, T lymphocyte defect and increase in urinary orotic acid, purine nucleoside phosphorylase (PNP)-deficiency was suspected. Gene targeted sequencing of the *PNP* gene showed a novel missense mutation in exon 5 (c.595A > C coding for the p.S199R substitution), inherited by the father who is heterozygous. The mother did not carry any evident variant in the gene. A chromosomal microarray analysis with a mean 100 Kb resolution was performed (Cyto-SNS850K) showed a de novo microdeletion encompassing 448 Kb in the 14q11.2 region, that comprises the *PNP* gene. The father did not carry this deletion. The p.S199R substitution is considered damaging in the Polyphen-2 software. The functional relevance of this genotype was confirmed by the marked reduction of PNP enzymatic activity (0.57 μmol/h/ml in the patient, normal value > 25 micromol/h/ml).

The patient received an haplohydentical hematopoietic stem cell transplantation (HSCT) from his mother with subsequent progressive clinical improvement. At last follow-up, 12 months after HSCT, he was in good clinical conditions, laboratory parameters were completely normal and his neurological impairment improved (Table 1).

Serum levels of IL-18 and of CXCL9, a chemokine directly induced by interferon-gamma (IFNγ), were markedly elevated at disease onset. They decreased progressively during disease course and normalized after the HSCT (Table 1).

## Discussion

We describe a patient with an onset highly suggestive for sJIA with MAS who was later diagnosed as having PNP-deficiency with HLH. Some unusual laboratory features that become apparent when HLH was controlled with glucocorticoids, i.e. persistent anaemia and persistent lymphopenia, led to additional investigations. While lymphopenia is usually present during MAS, it is not a feature of sJIA without MAS. Anaemia is typical of sJIA. The mean corpuscular volume was normal for age (ranging from 74 to 82 fL, with Hb ranging from 7.2 to 8.0 mg/dl), this being rather unusual for persistent severe anaemia associated with sJIA, that is usually microcytic [5]. Moreover, when the general conditions improved hypotonicity became evident. Altogether, persistent lymphopenia, severe normocytic anaemia and hypotonicity were not consistent with sJIA with MAS. The following investigations led to the diagnosis: low T cell count with defective response to mitogens suggestive of immunodeficiency, low haptoglobin with positive Coomb’s test suggestive of haemolytic anaemia and increased urinary orotic acid, a known intermediate metabolite of the pyrimidine biosynthetic pathway, suggestive of an alteration of purine salvage pathway. These findings were consistent with a diagnosis of PNP-deficiency.

PNP-deficiency is a rare autosomal recessive immunodeficiency caused by a mutation in the *PNP* gene that encodes for one of the enzymes involved in purine salvage [6, 7]. Defect in PNP leads to intracellular accumulation of metabolites, particularly deoxyguanosine triphostate (dGTP) which is toxic for neurons and T lymphocytes. PNP-deficiency is characterized by a progressive combined immunodeficiency, neurologic symptoms and autoimmune disorders. Patients present with bacterial, viral, and opportunistic infections. Approximately two-thirds of the patients have progressive neurologic symptoms, including developmental delay, muscle spasticity, ataxia, and pyramidal signs. The incidence of autoimmune disorders, including haemolytic anaemia, is increased [8–10]. Cases of HLH associated with PNP-deficiency have been reported [11].

Our patient fulfilled the HLH-2004 diagnostic criteria for primary HLH (5 out of 8 criteria, fever, cytopenia of two lines, increased ferritin, increased triglycerides and hemophagocytosis) [12]. Based on persistent fever, arthritis with tenosynovitis, rash and pericarditis, the presentation was consistent with sJIA. Duration of arthritis was shorter than 6 weeks, as per ILAR classification criteria [13], possibly due to rapid initiation of glucocorticoids because of overt HLH. He also met the classification criteria for MAS [14]. If we apply the MAS/primary HLH (MH) score, a score that may allow to distinguish patients with HLH from those with MAS during sJIA, our patient was more likely to have MAS [15].

Levels of IL-18 were markedly elevated (hundreds of ng/ml), in the range of those found in patients with MAS during sJIA in our lab (unpublished) and by Weiss et al. [16]. High IL-18 levels have been described in one patient with PNP-deficiency [17]. IL-18 is indeed known to induce IFNγ production [18] and IFNγ has been hypothesized to be a major pathogenic mediator of all forms of HLH [19]. High levels of IL-18 may therefore contribute to the predisposition of PNP-deficiency patients to develop HLH. Indeed, our patient had also high circulating levels of CXCL9, a chemokine induced by IFNγ, known to be significantly elevated in patients with all HLH forms, including MAS, and to be correlated with laboratory features of disease activity [16, 20, 21].

In summary, this patient had clinical and laboratory features, as well as elevated IL-18, consistent with sJIA and MAS. However, unusual clinical and laboratory findings suggested additional investigations that led to the diagnosis of PNP-deficiency mandating a significant change in therapeutic approach with urgent HSCT followed by prompt recovery. This case underlines the difficulties in discriminating sJIA from other causes of systemic inflammation. Furthermore, it supports the notion that, especially in young children with an sJIA-like disease, other mimicking conditions should be actively sought for, and underscores the need for in-depth longitudinal observations of clinical and laboratory features even when classification criteria for sJIA and for MAS complicating sJIA are met.

## Abbreviations

Anti-CD3: Cluster of differentiation 3
CRP: C reactive protein
CXCL9: (C-X-C motif) ligand 9
dGTP: Deoxyguanosine triphostate
ESR: Erythrocyte sedimentation rate
HLH: Haemophagocytic lymphohistiocytosis
HSCT: Hematopoietic stem cell transplantation
IFNγ: Interferon-gamma
IL-18: Circulating Interleukin-18
MAS: Macrophage activation syndrome
PBMCs: Peripheral blood mononuclear cells
PHA: Phytohemoagglutinin
pHLH: Primary or familial haemophagocytic; lymphohistiocytosis
PNP: Purine nucleoside phosphorylase
sHLH: Secondary or acquired haemophagocytic lymphohistiocytosis
sJIA: Systemic Juvenile Idiopathic Arthritis
